# The relationship between serum resolvin D1, NLRP3, cytokine levels, and adolescents with first-episode medication-naïve major depressive disorder

**DOI:** 10.1186/s12888-024-05724-0

**Published:** 2024-04-16

**Authors:** Jiamei Guo, Tanwei Zhang, Wanjun Chen, Jianyu Tan, Xiao Li, Anhai Zheng, Yixiao Fu, Tian Qiu

**Affiliations:** Department of Psychiatry, the First Affiliated Hospital of Chongqing Medical University, 400016 Chongqing, P.R. China

**Keywords:** Major depressive disorder, Adolescent, Resolvin D1, NLRP3, Cytokines, Medication-naïve

## Abstract

**Background:**

Inflammation has become a critical pathological mechanism of Major Depressive Disorder (MDD). NLRP3 is a critical inflammatory pathway to maintain the immune balance. Recently, preclinical evidence showed that Resolvin D1 might potentially offer a new option for antidepressant treatment due to its protective effects through the inhibition of neuroinflammation. However, whether they have clinical value in the diagnosis and treatment evaluation of adolescent depression was unclear.

**Methods:**

Forty-eight untreated first-episode adolescent patients with moderate to severe major depressive disorder, as well as 30 healthy adolescents (HCs, age and gender-matched), were enrolled for this study. Their ages ranged from 13 to 18 (15.75 ± 1.36) years. The patients were treated with fluoxetine for 6–8 weeks. HDRS-17 was used to evaluate the severity of depressive symptoms. Venous blood samples were collected at baseline for the two groups and at the time-point of post-antidepressant treatment for the patients. Serum concentrations of RvD1, NLRP3, IL-1β, IL-18, and IL-4 were measured by enzyme-linked immunosorbent assays (ELISA) pre- and post-fluoxetine treatment.

**Results:**

Serum levels of RvD1 and anti-inflammatory cytokine IL-4 were significantly elevated in adolescents with MDD compared to healthy adolescents, but no significant difference in NLRP3, IL-1β, and IL-18 between the two groups. Meanwhile, RvD1 (positively) and IL-4 (negatively) were correlated with the severity of symptoms (HDRS-17 scores) after adjusting age, gender, and BMI. Interestingly, fluoxetine treatment significantly reduced the serum levels of RvD1, NLRP3, IL-1β, and IL-18 in MDD adolescents but increased the levels of IL-4 relative to baseline. Furthermore, we observed that serum levels of RvD1 might be an excellent distinguishing indicator for depression and healthy adolescents.

**Conclusions:**

Our study is the first to compare RvD1 and NLRP3 between adolescent MDD and HCs. Our findings of reactive increase of RvD1 in adolescent MDD comprised a novel and critical contribution. Our results showed the presence of inflammation resolution unbalanced in adolescents with MDD and indicated that RvD1 might be an ideal biomarker for diagnosing and treating adolescent MDD.

## Background

Major Depressive Disorder (MDD) has become a significant public health issue, affecting approximately 350 million people’s health worldwide [1]. Adolescence is a peak period of onset for MDD onset. The prevalence of adolescent depression is higher than the general population, with an annual prevalence of 8% and a lifetime prevalence of 11% [2]. This problem has become the first burden of disease and the leading cause of self-injury and suicide in adolescents [3, 4]. Unfortunately, atypical clinical symptoms greatly complicate the early diagnosis of depression in adolescents [4]. Apart from this, response rates to treatment are much worse for psychotherapy and pharmacotherapy in adolescents than that in adults [5, 6]. Hence, a better understanding of the potential pathophysiological mechanisms associated with MDD in adolescents is crucial to improving available treatments’ effectiveness.

The exact pathogenesis of depression remains unclear. Previous studies on adolescent depression have primarily focused on serotonin and hypothalamic–pituitary–adrenal (HPA) axis dysregulation hypotheses [7, 8]. In recent decades, mounting evidence suggests that inflammation may play a significant role in depression, as evidenced by a higher prevalence of depression in patients with immune diseases, multiple sclerosis, and diabetes [9, 10]. Anti-inflammatory treatments, such as infliximab and minocycline, have also show significant antidepressant effects [11]. Elevated levels of peripheral cytokines were found in patients with depression [12]. Additionally, higher levels of pro-inflammatory cytokines are associated with disease severity, duration, and drug resistance [13, 14]. However, the role of inflammation in adolescent depression remains less studied, despite adolescence being a crucial period for immune system developments.

The Nod-like receptor family pyrin domain containing 3 (NLRP3), as a cytosolic sensor, is activated under a wide range of danger signals and is the critical marker to innate immune defense [15]. Once activated, it would induce the cleavage and secretion of the pro-inflammatory cytokines IL-1β and IL-18 and participate in the inflammation damage [16]. Pre-clinical studies show that the NLRP3 may be involved in depression by regulating the level of IL-1βin the serum and hippocampus. IL-1β is closely related to depression [17–19]. A clinical study observed increased gene expression of NLRP3 and caspase-1 in mononuclear blood cells of adult patients with MDD [20]. Recently, NLRP3 inflammasome activation was found related to mitochondrial health and white matter integrity in adolescent mood disorders [21]. However, whether gene expression of NLRP3 is still activated in the peripheral blood of adolescent patients with MDD, like adult depression, is still unclear.

Resolvin D1(RvD1) is one of the specialized pro-resolving mediators (SPMs), which docosahexaenoic acid synthesizes under the effect of 15-lipoxygenase and 5-Lipoxygenase [22]. SPMs enhance inflammation resolution and promote the repair of injured tissues. Effective resolution of inflammation is also critical to the self-limited inflammatory response [23]. Meanwhile, Unsaturated fatty acids and their metabolites, such as lipoxin, protectins, maresins, and resolvins, have been reported to fight against neuroinflammation and thus serve as powerful neuroprotectors [24]. Recent studies reported that SPMs might play an essential role in the clinical benefits of n-3 polyunsaturated fatty acids treatment in MDD [25]. Among the SPMs, RvD1 potentially offer a new option for antidepressant treatment. For example, it’s reported that the level of RvD1 in peripheral blood is significantly elevated in adult patients with bipolar disorder [26]. Pre-clinical studies have found that resolvin D1and D2 had a rapid positive effect on Lipopolysaccharide-induced or Chronic unpredictable mild stress depression-like behavior in rodents [27, 28]. Meanwhile, it has been shown to play an explanatory role by inhibiting NLRP3 inflammasome [29, 30]. However, until now, its role and the relationship with NLPR3 in adolescents with unipolar depression have not been explored.

We hypothesized that inflammation and pro-resolving mediator are involved in developing adolescent depression. RvD1, NLPR3, IL-1β, Il-18, and IL-4 might be related to the efficiency of fluoxetine (one of the antidepressant), which has been beneficial to adolescent depression among clinical common-use antidepressants [31]. Therefore, the first purpose of this study was to clarify the difference in peripheral RvD1, NLPR3, IL-1β, Il-18, and IL-4 between the adolescent patients with depression and the healthy control group. Next, we would longitudinally compare the changes in these five peripheral blood substances pre- and post-fluoxetine treatment. Thirdly, we would evaluate the predicted value of these five inflammatory markers for adolescent depression.

## Methods

### Participants

The Ethics Committee of the first affiliated hospital of Chongqing Medical University approved this study. A total of 130 adolescent patients diagnosed with moderate-severe major depressive disorder according to the Structured Clinical Interview for the DSM-V were recruited. only 48 of them who met the inclusion/exclusion criteria finished this longitudinal intervention follow-up study. All the patients were recruited from the outpatient department of psychiatry at the First Affiliated Hospital of Chongqing Medical University, China, from January 2021 to February 2022. The diagnosis was simultaneously made by two experienced and senior attending physicians. All enrolled patients met the inclusion and exclusion criteria below. The inclusion criteria were as follows: [1] patients’ ages ranged from 13 to 18 years old; [2] the first untreated episodes of depression; [3] with a score of the 17-item Hamilton Depression Rating Scale (HDRS-17)≥17. The exclusion criteria were as follows: [1] with a previous history of manic or hypomanic episodes; [2] having other mental, organic brain diseases; [3] accompanied by heart, liver, and kidney diseases, diabetes mellitus, and other somatic severe diseases; [4] a history of substance dependence or abuse (e.g., tobacco, alcohol, cocaine, drugs); [5] current use of any medication known to be likely to affect inflammation level, such as corticosteroids, anti-inflammatory medications, etc [6] having participated in other clinical trials in the previous eight weeks. A healthy control group (HCs, *n* = 30) with age-and sex-matched depression patients was recruited. All the control adolescents met the criteria as follows: [1] a score of HDRS-17＜8; [2] without a history of Axis I psychiatric disorders as assessed with a Structured Clinical Interview for the DSM; [3] without chronic or severe physical illness, pregnancy, and drug abuse; [4] no drug intake during the two weeks before the blood sampling. All patients were treated with the single drug of fluoxetine. The starting dose was 10-20mg/day. The amount might be increased to 30-40mg/day according to the patient’s severity of depressive symptoms during follow-up. Primary demographic data of all participants were recorded, and HDRS-17 was used to assess the severity of the depressive symptoms. Written informed consent was obtained from participants’ parents or legal guardians.

### Serum collection

Venous blood samples were collected at baseline (patients and HCs) and after 6–8-week fluoxetine treatment (only patients). Four milliliters of venous blood were drawn by venipuncture into an anticoagulant-free vacuum tube from each participant after 8–10 h of fasting. The blood was centrifuged at 3,000 rpm for 5 min after standing for 1–2 h at room temperature. The isolated serum samples were transferred into the sterile Eppendorf tubes and stored at − 80 °C. Serum levels of RvD1, NLRP3, IL-1β, IL-18, and IL-4 were measured by Human RvD1 ELISA Kit (ED-11,506, LCS Ltd., China), Human NLRP3 ELISA kit (ED-11,695, LCS Ltd., China ), Human IL-1βELISA kit (ED-10,351, LCS Ltd.,China), Human IL-18 ELISA kit ( ED-10,349, LCS Ltd., China) and Human IL-4 ELISA kit (ED-10,375, LCS Ltd., China) according to the manufacturer’s recommendations.

### Statistical analysis

Data were analyzed in IBM SPSS Statistics 22.0. Categorical variables were expressed as numbers, and continuous variables were expressed as the mean ± standard deviation (M ± SD). The Shapiro-Wilk and Levene tests were used to test normal distribution and homogeneity of variance, respectively. Two samples T-test or the Mann-Whitney test, were used to compare the difference in the five inflammatory markers between the MDD and Control groups, as appropriate. Paired t-test or related Mann-Whitney test was performed to measure changes in the five inflammatory markers in the MDD group pre-and post-treatment. We also analyzed Pearson correlations and linear regression analyses in RvD1, NLPR3, several cytokines, and the severity of depression. ROC curve analysis predicted the differentiated power for MDD patients and healthy adolescents. *P* (two-tailed) < 0.05 was considered statistically significant.

## Results

### Clinical outcomes

The demographics of the participants are shown in Table 1. There was a significant difference in HDRS-17 depression scores between the adolescent depression group and the normal control group, but no significant differences in age, sex, and BMI (*P* > 0.05), suggesting that the two groups were comparable to a certain extent. (Table 1).

**Table 1:** Demographic and clinical data

	Depressive adolescents (*n* = 48)		Healthy control (*n* = 30)	t/F	P-value
Age, x ± s	15.75 ± 1.36		15.73 ± 1.55	0.05	0.96
gender(M/F)	18/30		13/17	0.262	0.641
BMI, x ± s	21.05 ± 3.78		20.04 ± 3.41	1.193	0.237
HDRS, x ± s	25.58 ± 5.90		2.53 ± 2.39	24.106	<0.001*

For continuous variables, statistical analysis was performed by Student’s T-test. For categorical variables, *P* -value was calculated using the chi-square test. *Statistically significant: *P* < 0.05

### Serum RvD1, NLRP3, and cytokines comparisons at baseline between the adolescent patients with MDD and HCs

Differences in serum levels of RvD1, NLRP3, IL-1β, IL-18, and IL-4 were evaluated between depressed adolescents and healthy controls. As shown in Table 2, the levels of RvD1 (*P* < 0.001) and IL-4 in depressed adolescents were significantly higher than those in the HCs group (Table 2). However, the two groups had no significant differences in the levels of NLRP3, IL-1β, and IL-18 (*P* > 0.05). It suggests that the serum levels of RvD1 and cytokine levels (especially anti-inflammatory cytokine IL-4) may be associated with adolescent depression.

**Table 2:** Serum RvD1, NLRP3, and cytokines comparisons between adolescent MDD and the HCs

	Depressive adolescents (*n* = 48)		Healthy control (*n* = 30)	t/Z	P-value
RvD1(pg/ml)	400.83 ± 91.83		290.91 ± 76.21	5.479	<0.001*
NLRP3(pg/ml)	915.65 ± 148.13		887.96 ± 156.44	0.786	0.434
IL-1β(pg/ml)	57.08 ± 11.96		57.70 ± 11.68	-0.216	0.829
IL-18(pg/ml)	261.79 ± 54.59		265.78 ± 40.90	-0.369	0.713
IL-4(pg/ml)	31.98 ± 4.87		29.00 ± 5.60	2.407	0.019*

For continuous variables, data were presented as M ± SD. *P*-value was calculated using an independent sample T-test for normally distributed or the Mann-Whitney nonparametric test for nonnormally distributed

*Statistically significant: *P* < 0.05

### Serum RvD1, NLRP3 and cytokines comparisons between pre- and post-fluoxetine treatment

As shown in Table 3, after 6–8 weeks of fluoxetine treatment, the total scores on the HDRS-17 were significantly decreased (*P* < 0.05), but still higher than the healthy control group (2.53 ± 2.39). The RvD1, NLRP3, IL-1β, IL-18, and IL-4 serum levels were dynamically evaluated before and after 6-8-week anti-depression treatment. After fluoxetine treatment, the serum levels of RvD1, NLRP3, and IL-1β in adolescent patients with MDD were significantly decreased (*P* < 0.01), whereas IL-4 was significantly increased (*P* < 0.001) compared with that before treatment (*P* < 0.001). However, there is no significant difference in the level of IL-18 between pre- and post-treatment.

**Table 3:** Levels of RvD1 and cytokine in adolescent patients with MDD after treatment

	Pre-MDD (*n* = 48)		Post-MDD (*n* = 48)	t/Z	P-value
HDRS	25.58 ± 5.90		14.48 ± 6.14	9.217	<0.001*
RvD1 (pg/ml)	400.83 ± 91.83		340.89 ± 82.00	5.768	<0.001*
NLRP3 (pg/ml)	915.65 ± 148.13		766.08 ± 184.60	4.657	<0.001*
IL-1β (pg/ml)	57.08 ± 11.96		46.21 ± 9.87	3.627	0.001*
IL-18 (pg/ml)	261.79 ± 254.59		254.59 ± 41.77	0.661	0.512
IL-4 (pg/ml)	31.98 ± 4.87		38.52 ± 6.87	-5.289	<0.001*

For continuous variables, data were presented as M ± SD. *P*-value was calculated using the paired t-test for normally distributed or the Mann-Whitney paired nonparametric test for nonnormally distributed

*Statistically significant: *P* < 0.05

### Correlation between the severity of depressive symptoms and serum levels of RvD1, NLRP3, IL-1, IL-18, and IL-4 in adolescent patients with depression

We further analyzed the correlations between the severity of the depressive symptom and the serum levels of RvD1, NLRP3, IL-1β, IL-18, and IL-4. The Pearson correlation analysis is shown in Table 4. For pre-and post-treatment, the HDRS scores were positively correlated with RvD1 (*r* = 0.310, *P* = 0.002), NLRP3 (*r* = 0.271, *P* = 0.008), IL-18 (*r* = 0.257, *P* = 0.012), and IL-1βlevels (*r* = 0.286, *P* = 0.008), and the HDRS scores were negatively correlated with IL-4 levels (*r* = -0.331, *p* = 0.002). NLRP3, IL-1βand IL-4 were also related to each other.

Linear regression analysis showed the serum levels of resolvinD1 (standardized β = 0.233, *P* = 0.036) was positively related to the scores of HDRS-17 and serum levels of IL-4 (standardized β = -0.329, *P* = 0.005) were negatively associated with the score of HDRS after adjusted the age and BMI (Table 5).

**Table 4:** The Pearson correlation analysis among depressive symptom severity and levels of RvD1 and cytokines

	HDRS	RvD1	NLRP3	IL-1β	IL-18	IL-4
HDRS	1	-	-	-	-	-
RvD1	0.310**	1	-	-	-	-
NLRP3	0.271**	0.267**	1	-	-	-
IL-1β	0.286**	0.201	0.257*	1	-	-
IL-18	0.257*	0.149	0.012	0.166	1	-
IL-4	-0.331**	0.021	0.407***	-0.269*	-0.136	1

* Statistically significant: *P* <0.05; ** Statistically significant: *P* <0.01;*** Statistically significant: *P* <0.001

**Table 5:** Multiple linear regression analysis among depressive symptom severity and levels of RvD1 and cytokines

Predictors	Unstandardized B	SE	Standardized β	T	P value
RvD1	0.021	0.010	0.233	2.134	0.036*
NLRP3	-0.002	0.005	-0.045	− 0.400	0.690
IL-1β	0.098	0.068	0.152	1.439	0.154
IL-18	0.016	0.018	0.100	0.890	0.376
IL-4	-0.381	0.133	-0.329	-2.865	0.005*

After adjusting for age and BMI

*Statistically significant: *P* < 0.05

### Differentiated values of RvD1 and IL-4

**Fig. 1:**
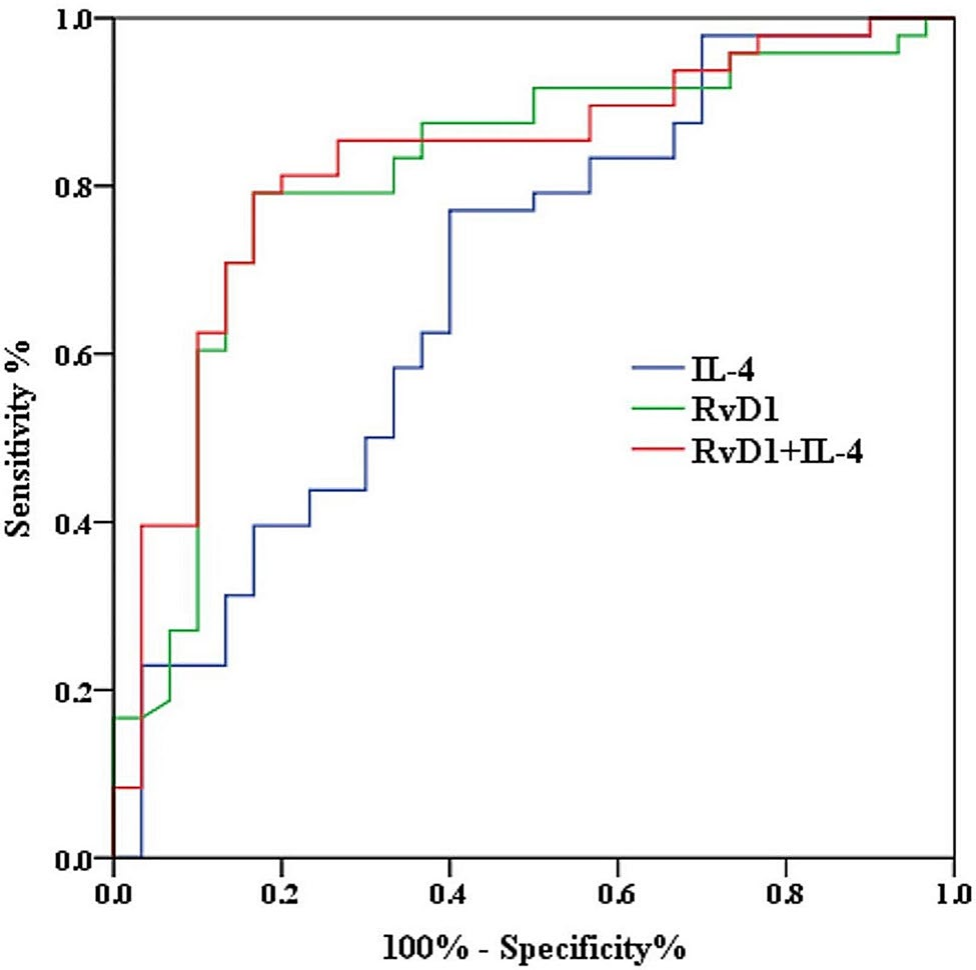
The area under the curves (AUCs) between adolescence MDD and HC reflect the differentiated power of RvD1, IL-4 and combination of the two indicators (IL-4: AUC = 0.684, *P* = 0.006; RvD1: AUC = 0.812, *P* < 0.001; IL-4 + RvD1: AUC = 0.825, *P* < 0.001;)

ROC curve analysis predicted the differentiated power for MDD patients and healthy adolescents. RvD1 and IL-4, which differed significantly between the two groups (adolescent MDD and HCs) and were independently associated with the severity of depressive symptoms, were chosen as potential predictors. The ROC curve is shown in Fig. 1. The AUCs, specificity, and sensitivity of RvD1 for identifying adolescent MDD and HC were 0.812, 83.7%, and 79.2%; the cut-off value was 318.04 pg/ml. The AUCs, specificity, and sensitivity of IL-4 were 0.684, 60%, and 77.1%; the cut-off value was 30.02 pg/ml. The AUCs, specificity, and sensitivity of the combinations of the two predictors were 0.825, 83.3% and 79.2%, almost equal to the indicator of RvD1.

## Discussion

In this study, we mainly observed increased levels of RvD1 and anti-inflammatory cytokine IL-4 in the peripheral blood of MDD adolescents compared to healthy adolescents. And, fluoxetine treatment reversed the increase of serum RvD1 in adolescent patients with MDD, significantly decreased the levels of pro-inflammatory cytokine IL-1β and NLRP3, while significantly increased the levels of anti-inflammatory cytokine IL-4 compared to baseline. Secondly, we also found that the levels of RvD1 were positively and IL-4 were negatively correlated with the severity of primary untreated adolescent depression. Thirdly, we presented that peripheral RvD1 might be an excellent distinguishing indicator for depression and healthy adolescents. This is the first study to examine the relationship between peripheral RvD1 and depression in adolescents. Our findings revealed that SPMs (at least RvD1) might play an essential critical role in the pathophysiological mechanism of adolescent depression, and the clinical value of RvD1 may be greater than that of cytokines or inflammatory targets, such as NLRP3, IL-1β, IL-18 and IL-4 in this study.

The finding of elevated RvD1 in adolescent MDD was consistent with the results reported in the previous clinical study, in which they found that serum RvD1 levels were significantly higher in the manic and depressive episodes of male adult patients with bipolar disorder in comparison with the controls [26]. Until now, studies on RvD1 in clinical neuropsychiatry are minimal. One multiple sclerosis (MS) studies also represented increased RvD1 in Cerebrospinal Fluid patients with higher disease activity [32]. However, decreased RvD1 in patients with ischemic stroke was also reported [33]. Two types of responders to inflammatory responses exist in humans. One type is characterized by an acute inflammatory reaction, followed by “early regression,” and the other type is characterized by the gradual increase of inflammation over time, followed by “delayed regression.“ [34]. Correspondingly, two groups of resolvers were proposed during the inflammation resolution.

The ‘early resolvers’ induced by rapid leukocyte accumulation and cytokine/chemokine synthesis were low initially and gradually increased as inflammation was alleviated. The ‘delayed resolvers’ caused by a gradual increase in inflammatory response were high early in the reaction but decreased as the inflammation progressed [34]. Depression is not an acute infectious disease; inflammatory responses gradually increase under long-term mental and psychological stress [35]. “Delayed resolution” and ‘delayed resolvers’ might be induced by this inflammatory response. Hence, we speculated that a reactive increase in RvD1, which serves as a ‘delayed resolver,’ might be a response to the inflammation in the first episode of adolescent depression.

Activation of NLRP3 and increased secretion of IL-1β and IL-18 are essential pathophysiological mechanisms of depression [16]. Effective inhibition of NLRP3 activation and reducing the secretion of related cytokines can improve depressive symptoms [36]. In this study, we also observed a significant decrease in NLRP3 and IL-1β after fluoxetine treatment in adolescent MDD relative to baseline. In general, the evidence for inflammation in adolescent depression does differ dramatically from that in adult depression. Most studies on adult depression and inflammation have reported consistent evidence of elevated pro-inflammatory factors [37]. However, most case-control studies reported no significant difference in peripheral IL-1βbetween adolescents with depression and healthy controls [38–40], which is consistent with our research. Only a few studies have found elevated peripheral cytokines in adolescents with depression [41, 42]. Compared to common cytokines like INF-γ, IL-6, and TNF-a, NLRP3 and IL-18 have rarely been studied in clinical studies of mental disorders. Luo et al. reported that serum IL-18 levels were significantly increased only in depressive states of bipolar disorder patients but not in manic and mixed states [43]. Wedervang-Resell et al. also found serum levels of IL-18 were significantly elevated in patients with early onset psychosis (aged 12–18 years) [44]. To our knowledge, they have not been explored in adolescents with depression until now. There was also inconsistent evidence on peripheral IL-1βchanges in adolescents with depression after treatment. For example, Pérez-Sánchez et al. reported that the pro-inflammatory cytokines (IFN-γ, IL-1, TNF-α, IL-6, IL-12, and IL-15) were decreased, and anti-inflammatory cytokines IL-2, IL-4, and IL-5 were increased after fluoxetine treatment in comparison with baseline [42], which was consistent with our finding. But Amitai et al. reported that antidepressant treatment only significantly reduced TNF-α levels, while IL-6 and IL-1β levels did not change significantly [40]. The above Inconsistent findings regarding inflammatory factors and adolescent depression may be partial attributed to different study designs, small sample sizes, heterogeneity of depression, variability in the dose and duration of treatment. We suspected that the non-significant increase in serum NLRP3 in adolescent depression patients may need to be considered the following factors: (1) the main location of the NLRP3 is intracellular, the inflammation in adolescents might not be severe enough to induce significant cellular damage for enhanced NLRP3 exposure or secretion; (2) Other psychiatric pathology studies revealed differences when examining NLRP3 in monocellucur blood cells via Western blot or Polymerase Chain Reaction, the method used in our study might not be sensitive to detect the NLRP3 expression.

In addition, specialized pro-resolving mediators are a class of molecules that have anti-inflammatory properties and promote the repair of damaged tissue, which are closely related to inflammatory signaling pathways and cytokines [45]. Strong evidence shows that RvD1 can inhibit the initiation and activation of NLRP3 and decrease the production of related cytokines. This protective role of RvD1 has been reported in the disease of diabetic retinopathy [46], cerebral ischemia/reperfusion injury [30], and spinal nerve ligation (SNL)-induced neuropathic pain [47]. In this study, we found that in the first-episode untreated MDD patients, the levels RvD1 and IL-4 were increased, and the pro-inflammatory factors (NLRP3, IL-1β,IL-18) were not significantly increased. After antidepressant treatment, with the RvD1 consumption, the levels of NLRP3 and IL-1βsignificantly decreased, while the level of IL-4 was further increased compared to that before treatment. Hence, we assumed reactive increase in RvD1 might have an anti-inflammatory effects by inhibiting NLRP3 expression and anti-inflammatory factor IL-1βsecretion, and promoting anti-inflammatory factors secretion (IL-4).

Furthermore, our study also found that serum levels of IL-4 were negatively correlated with the severity of adolescent depression. A higher level of IL-4 might be a protective factor for adolescent MDD. The finding is consistent with the studies reported by Pérez-Sánchez [42]. However, there are many inconsistencies in clinical studies on peripheral IL-4 levels between adolescent depression and healthy control. Some studies reported elevated levels of IL-4 in the peripheral blood of adolescents with depression [48] compared to healthy adolescents. Gabbay et al. found no significant difference in the IL-4 between adolescents with MDD and healthy adolescents [49]. Even some studies showed a reduction of IL-4 in adolescent patients with depression. The reduction was even more significant in adolescent depression with suicidal ideation/behavior [50]. IL-4, a type of Th2 cytokines with anti-inflammatory effects, has been widely reported to have a protective effect on depression in pre-clinical studies [51, 52]. In studies of adult depression, more evidence has also been informed of elevated levels of IL-4 in peripheral blood [53, 54]. In our study, the significant increase of IL-4 in the peripheral blood of adolescents with depression and further increase after fluoxetine treatment may confirm the protective role of IL-4 in the pathophysiological process of adolescents with depression.

However, our study also had some limitations, such as a small number of subjects, a relatively high proportion of female patients, and insufficient observation time points. In addition, we only detected peripheral RvD1, NLRP3, and several common cytokines, without exploring other cytokines which have been reported to participate in the development of depression. Furthermore, our study did not detect central inflammation, so the results could not explain the association between peripheral inflammation and central inflammation. Hence, in future studies, we would expand the sample size, including more demographic data, distinguish different subgroups, and add more treatment time points to provide more reliable evidence for SPMs involved in the pathophysiological mechanism of adolescent depression.

## Conclusion

Adolescence depression is a complex and highly heterogeneous disease. Inflammation and alterations in circulatory cytokines play roles in the development of MDD in adults as well as in adolescents. However, more inconsistent findings on which inflammatory markers are abnormal in adolescents with MDD. To some extent, our findings confirmed the role of pro-resolving lipid mediators and cytokines in the pathophysiological mechanism of adolescent depression. More importantly, results showed that RvD1 might be another more promising biomarker for the diagnostic and treatment assessment of adolescent MDD.

## Data Availability

The original contributions presented in this study are included in the article. All data contained in this study are available on reasonable request to the corresponding author.
